# Evaluation of base materials of TL slab dosimeter for heavy-ion radiotherapy

**DOI:** 10.1093/jrr/rrt186

**Published:** 2014-03

**Authors:** Yusuke Koba, Kiyomitsu Shinsho, Satoshi Tamatsu, Shigekazu Fukuda, Genichiro Wakabayashi

**Affiliations:** 1 National Institute of Radiological Sciences, 4-9-1, Anagawa, Inage-ku, Chiba 263-8555, Japan; 2 Tokyo Metropolitan University, Tokyo, Japan; 3 Chiba University, Chiba, Japan; 4 Kinki University Atomic Energy Reseach Institute, Osaka Japan

**Keywords:** TLD, TL slab dosimeter, heavy-ion radiotherapy, three-dimensional dosimetry

## Abstract

In order to measure a three-dimensional dose distribution in X-ray radiotherapy, we developed TL slab dosimeter with new TL phosphor Li_3_B_7_O_12_(Cu), which has *Z*_eff_ = 7.42 and a density of 1.01 g/cm^3^ and synthetic resin as binder [ 1]. We can measure a three-dimensional dose distribution easily and reliably using this detector. This detector showed a promising tool for QA/QC in advanced X-ray radiotherapies such as IMRT, etc. In heavy-ion radiotherapies which shape precipitous dose distributions, it is also necessary to measure three-dimensional dose distribution easily. To use TL slab dosimeter in heavy-ion dosimetry, it is essential to measure its LET dependence sufficiently. And it is necessary to evaluate the dosimetric water equivalence of this dosimeter for heavy ions. Previous studies showed that the relative TL efficiency of this TL phosphor decreased to ∼20% at the Bragg-peak of carbon 290 MeV/u beams and the stopping-power ratio of this dosimeter to water for carbon ions was 0.87 [ 2]. These results were not good for application in heavy-ion radiotherapy. It was often reported that there is a relationship between the glow curve shape of general TLDs (such as LiF and BeO) and LET. Using this relationship of glow curve and LET, the relative TL efficiency can be corrected and we could apply TLDs to dose measurement in heavy-ion radiotherapies.

In this study, in order to develop better TL slab dosimeter for heavy-ion radiotherapy using TL phosphors with the above characteristics, we evaluated the dosimetric water equivalence of several base materials for TL slab dosimeter. We chose several kinds of ceramics with heating resistance as the base material; ISOPLATON E3, P1, M2, A98 S1 and Machinable Ceramics, TBS N64, N66, N1, N3 (ISOLITE Co., Ltd). We focused attention on stopping power, scattering power and nuclear cross-section of these materials for heavy ions. We calculated these interactions using the Bethe formula, the Gottschalk formula and the Sihver formula. Figure 1 shows the result of theoretical calculation of the water-equivalence ratio, stopping power ratio *S*/*S*_w_, scattering power ratio *T*/*T*_w_ and nuclear cross-section ratio σ/σ_w_ for carbon-ion beam. In these ceramics, ISOPLANTON S1 was the best of base material for TL slab dosimeter for carbon-ion beam. On the basis of this evaluation, we will develop the TL slab dosimeter for heavy-ion radiotherapy.

**Fig. 1.**
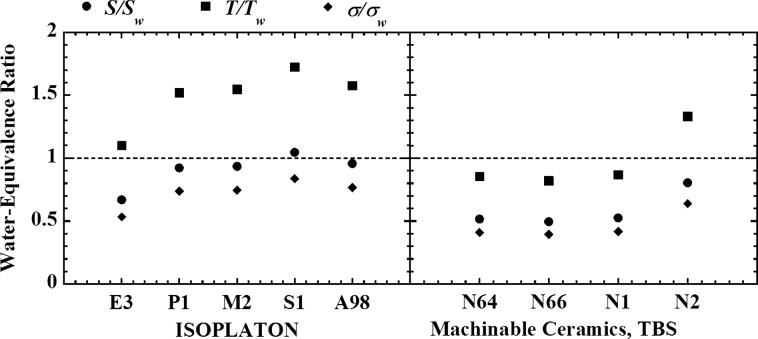
Water-equivalence ratio of the base materials for TL slab dosimeter.

Water-equivalence ratio of the base materials for TL slab dosimeter.
